# Virus-like particles vaccine containing *Clonorchis sinensis* tegumental protein induces partial protection against *Clonorchis sinensis* infection

**DOI:** 10.1186/s13071-017-2526-5

**Published:** 2017-12-29

**Authors:** Dong-Hun Lee, Ah-Ra Kim, Su-Hwa Lee, Fu-Shi Quan

**Affiliations:** 1 Department of Biomedical Science, Graduate School, Kyung Hee University, Seoul, South Korea; 2 Department of Medical Zoology, Kyung Hee University School of Medicine, Seoul, South Korea

**Keywords:** *Clonorchis sinensis*, Virus-like particles, Vaccine, Protection

## Abstract

**Background:**

Human clonorchiasis, caused by the infection of *Clonorchis sinensis*, is one of the major health problems in Southeast Asia. However, vaccine efficacy against *C. sinensis* infection remains largely unknown.

**Methods:**

In this study, for the first time, we generated virus-like particles (VLPs) vaccine containing the *C. sinensis* tegumental protein 22.3 kDa (CsTP 22.3) and the influenza matrix protein (M1) as a core protein, and investigated the vaccine efficacy in Sprague-Dawley rats.

**Results:**

Intranasal immunization of VLPs vaccine induced *C. sinensis*-specific IgG, IgG2a and IgG2c in the sera and IgA responses in the feces and intestines. Notably, upon challenge infection with *C. sinensis* metacercariae, significantly lower adult worm loads (70.2%) were measured in the liver of rats immunized with VLPs, compared to those of naïve rats. Furthermore, VLPs immunization induced antibody secreting cells (ASC) responses and CD4+/CD8+ T cell responses in the spleen.

**Conclusions:**

Our results indicated that VLPs vaccine containing *C. sinensis* CsTP 22.3 kDa provided partial protection against *C. sisnensis* infection. Thus, VLPs could be a potential vaccine candidate against *C. sinensis*.

**Electronic supplementary material:**

The online version of this article (10.1186/s13071-017-2526-5) contains supplementary material, which is available to authorized users.

## Background

Human clonorchiasis is one of the most important food-borne zoonosis, caused by *Clonorchis sinensis* infection from the consumption of raw or undercooked freshwater fish infected with *C. sinensis* metacercariae. Clonorchiasis is mainly prevalent in Southeast Asia, including Korea, China, East Russia, Taiwan and northern Vietnam, causing pyogenic cholangitis, cholelithiasis, cholecystitis and hepatic fibrosis, and even cholagiocarcinoma in humans [1–4]. Thus, *C. sinensis* has been classified as a group 1 biocarcinogens by the International Agency of Cancer Research, and clonorchiasis is included in control program of neglected tropical diseases by WHO [2, 5]. It is estimated that 200 million people are at risk of infection, 15–20 million people are infected with *C. sinensis* worldwide and, among them, 1.4 million people are currently infected with this fluke in South Korea [2, 6]. The development of vaccines would have a significant impact towards the ultimate goal of disease elimination.

Vaccines against *C. sinensis* infection are largely unknown. No commercially produced vaccine is yet available for the prevention of *C. sinensis* infection. Irradiated metacercariae of *C. sinensis* have been reported to generate resistance to infection [7]. Intramuscular injection of a plasmid containing genes encoding cysteine proteinase, fatty acid-binding protein, CsPMY and enolase (CsENO) elicited worm reductions, rating 31.50, 40.90, 31.60 and 37.42%, respectively [8, 9]. Subcutaneous inoculation with recombinant proteins Rho GTPase, 14-3-3 epsilon, CsPMY, cathepsin B cysteine protease 2 (CsCB2), CsCB3, CsENO and hexokinase (CsHK), showed worm reduction rates of 60.4, 45.38, 54.30, 41.00, 67.00, 56.29 and 50.20%, respectively [9–13]. Oral delivery of *Bacillus subtilis* spores expressing a 22.3 kDa tegumental protein of *C. sinensis* and CsENO elicited 44.70 and 60.07% of worm reduction, respectively [14, 15]. Taken together, such vaccine efficacies are limited and not well suited for field use.

Virus-like particles (VLPs) are recombinant vaccines, displaying promising results in preclinical and clinical studies in terms of both safety and efficacy [16]. VLPs are morphologically similar to live viruses, but lack viral genetic materials and therefore cannot replicate, which is advantageous for safety [17–19]. In this study, we, for the first time, generated VLPs vaccine containing *C. sinensis* tegumental protein (CsTP22.3). We found that *C. sinensis* VLPs vaccine elicited *C. sinensis*-specific IgG, IgG subclass and IgA antibody responses, antibody secreting cells (ASC) and CD4+/CD8+ T cell responses, resulting in protection against *C. sinensis* infection in a rat model.

## Methods

### Cells, viruses, parasites, antibodies and animals

*Spodoptera frugiperda* SF9 insect cells were maintained in suspension in serum-free SF900II medium (Invitrogen, Carlsbad, USA) at 27 °C. HEp-2 cells were obtained from ATCC. HEp-2 cells were grown in tissue culture flasks in Dulbecco’s modified Eagle medium (DMEM) with 10% fetal bovine serum (FBS), penicillin and streptomycin at 37 °C with 5% CO_2_. MDCK cells were infected with influenza virus (A/California/04/09) to obtain influenza virus total RNA. Monoclonal mouse anti-RSV fusion protein (131-2A) (Millipore, Burlington, USA) was used in virus plaque assay. Mouse monoclonal antibody to influenza A virus M1 (Abcam, Cambridge, UK) was used in western blot. HRP-conjugated goat anti-rat immunoglobulins G (IgG), IgG1, IgG2a, IgG2b and IgG2c (Southern Biotech, Birmingham, USA) were used for secondary antibodies. Sprague-Dawley (SD) rats (female, 8 weeks old) and New Zealand white rabbits (male, 2–4 month old) were purchased from Samyook Animal Center, Osan City, Kyonggi-do, Korea. White rabbits were infected with *Clonorchis sinensis* metacercariae to generate *C. sinensis* adult worms. *Clonorchis sinensis* metacercariae were collected from the freshwater fish *Pseudorasbora parva* by digesting muscles with pepsin-HCl, followed by filtration through layers of gauze.

### Preparation of *C. sinensis* antigen

*Clonorchis sinensis* were collected from rabbit liver and the excretory-secretory antigen (ES Ag) of *C. sinensis* obtained as described previously [20]. *Clonorchis sinensis* adults were cultured in RPMI 1640 medium supplemented with antibiotics at 37 °C in the presence of 5% CO_2_. Culture medium was collected and centrifuged at 4 °C and 1000× *rpm* for 30 min, and the supernatants were lyophilized at -20 °C. Protein concentration was determined, and samples were stored at -70 °C until use.

### Construction of recombinant baculovirus (rBV) expressing *C. sinensis* tegumental protein (CsTP22.3) or influenza M1

Total RNA was extracted from *C. sinensis* adults using RNeasy Mini Kit (Qiagen, Valencia, USA) and complementary DNA (cDNA) was synthesized. The RNA was reverse transcribed to cDNA using a Prime Script 1st strand cDNA synthesis kit according to the manufacturer’s instructions (Takara, Otsu, Japan). cDNA was used as a template to amplify the complete coding sequence of CsTP22.3 by polymerase chain reaction (PCR). The primers were designed according to the nucleotide sequence of CsTP22.3 in GenBank (accession number: EF077216.1): forward (5′-AAA GAA TTC ACC ATG TGC GCA CTT TCC TCA CA-3′) and reverse (5′-TTA CTC GAG TCA TAC CCA CGG TGT CTT CCA-3′) with *EcoR*I and *Xho*I restriction enzyme sites (underlined). PCR products were inserted into the pFastBac vector (Invitrogen, Carlsbad, USA). For influenza M1 gene cloning, all procedures were followed as described previously [21]. The recombinant plasmid was transformed into *E. coli* DH5-alpha and transferred into a DH10-Bac. All clones were confirmed by DNA sequencing and recombinant plasmid DNAs were stored at -20 °C until used.

### Generation of rBV and virus-like particles (VLPs)

Generation of recombinant baculovirus (rBV) was obtained using a Bac-to-Bac expression system (Invitrogen), according to the manufacturer’s instructions. Briefly, DNA plasmids containing CsTP 22.3 or M1 genes were transfected into SF9 cells using cellfectin II (Invitrogen). VLPs were produced by co-infecting SF9 cells with rBV expressing CsTP22.3 and influenza matrix 1 (M1). To collect the supernatants, cell culture was centrifuged at 6000 rpm for 20 min at 4 °C 2–3 days post-infection. The supernatant was pelleted using ultra-centrifugation. The pelleted VLPs were purified by 20–30–60% sucrose gradient at 30,000× *rpm* for 1 h at 4 °C. The VLP bands between 30 and 60% were collected and pelleted at 28,000× *rpm* for 40 min at 4 °C. To re-suspend the VLPs, they were incubated in PBS overnight at 4 °C. Protein concentration was determined using BCA Assay Kit (Sigma-Aldrich, St. Louis, USA).

### Characterization of VLPs

Western blots and electron microscopy were used to characterize the VLPs. For western blot analysis, rat serum that was infected with *C. sinensis* was used to probe CsTP 22.3 protein. For M1 protein detection, monoclonal mouse anti-M1 antibody was used. HRP-conjugated goat anti-mouse IgG was used as secondary antibody. VLPs were negatively stained using phosphotungstic acid (pH 7.0) and transmission electron microscopy (JEOL 2100, JEOL USA, Inc.; Peabody, MA, USA) was performed at 200 kV to characterize the VLP morphology [22].

### VLPs immunization, challenge infection and sample collection

Female Sprague-Dawley rats aged 8 weeks were divided into 3 groups: naïve control, *C. sinensis* infection control (Naïve + Cha) and VLP immunized plus challenge infected (Immu + Cha). Each group contained 12 rats. Rats were immunized intranasally twice with VLPs (200 μg/rat) at 4-week intervals. At 4 weeks after boost immunization, naïve or immunized rats were challenge infected with 50 *C. sinensis* metacercariae. Blood samples were collected by puncture of the retro-orbital plexus at 1 week and 4 weeks after prime, boost and challenge infection. Serum samples were collected and stored at -20 °C. Feces and intestines were collected at 1 or 4 weeks after challenge infection. Individual rat feces, liver and small intestines were collected as described previously [20]. The collected intestine was incubated in 0.85% saline at 37 °C for 1 h. The intestinal mucus was collected and centrifuged at 2000× *rpm* for 10 min. The supernatant was stored at -70 °C until use. The experiment was repeated twice.

### Antibody response by ELISA

*Clonorchis sinensis*-specific antibodies (IgG, IgG1, IgG2a, IgG2b and IgG2c) were determined in sera and *C. sinensis*-specific IgA antibody in feces, liver and intestines by enzyme-linked immunosorbent assay (ELISA). Briefly, 96-well plates were coated with 4 μg/ml of *C. sinensis* ES antigen at 4 °C overnight. *Clonorchis sinensis* ES antigen was found to contain CsTP22.3 by SDS-PAGE (Additional file 1: Figure S1a). The plates were washed and then blocked with 0.2% gelatin in PBST for 2 h at 37 °C. After washing, diluted sera (1:100), feces (1:50) or intestine (1:50) samples were added and incubated for 2 h at 37 °C. Antibody responses were detected using the HRP-conjugated goat anti-rat secondary antibody (IgG, IgG1, IgG2a, IgG2b and IgG2c). The substrate *O*-phenylenediamine in citrate-phosphate buffer (pH 5.0), containing 0.03% H_2_O_2_, was used to develop color and stopped with 2N H_2_SO_4_. The optical density at 490 nm was measured with an ELISA reader.

### Analysis of antibody-secreting cell response in vitro

*Clonorchis sinensis*-specific antibody-producing cells were determined in the spleen in vitro as described previously [20]. HRP-conjugated secondary goat anti-rat antibodies (IgG, IgG1, IgG2b, and IgG2c) were used and the substrate *O*-phenylenediamine (Zymed, San Francisco, USA) was used; the optical density was measured at 490 nm. The levels of parasite-specific antibodies secreted into the culture media and bound to the coated antigens were determined as previously described [21].

### Flow cytometry analysis

To perform cell phenotype analysis, single cell suspensions from the spleen were isolated from homogenized tissues. For cell phenotype analysis, 1 × 10^6^ splenocytes were stained with surface marker antibodies including CD3e-PE-Cy5, CD4-FITC, CD8a-PE (BD Biosciences, Franklin Lakes, USA). Stained cells were acquired on a BD FACSCalibur and BD AcuriC6 and analyzed using Flow Jo software and BD AcuriC6 software.

### Liver histopathology

Individual liver lobes harvested from rats at week 4 post-challenge were inflated and fixed with 10% neutral buffered formalin. The liver tissues were embedded in paraffin, sectioned and stained with hematoxylin and eosin (H&E, 200× magnification) to assess histological changes as described [23]. At least eight sections per rat were obtained for histopathology analysis.

### Worm burden counts in the liver

Rats were challenged with 50 *C. sinensis* metacercariae and after 1 month flukes were recovered from the bile duct of the rats and counted as described previously [20, 24]. The protection rate was calculated as described previously [25].

### Statistics

All parameters were recorded for individuals within groups. Statistical comparisons of data were carried out using the Student’s t-test and one-way ANOVA of PC-SAS 9.3. *P-*values < 0.05 were considered to be significant.

## Results

### Constructs generation

*Clonorchis sinensis* CsTP 22.3 gene was amplified by PCR and the influenza M1 gene was amplified by RT-PCR with primers containing restriction enzyme sites (Additional file 2: Figure S2a, c). Genes were cloned into pFastBac vectors, and insertion of CsTP 22.3 and M1 in pFastBac expressing vectors was confirmed by cutting with restriction enzyme sites, CsTP 22.3: *EcoR*I and *Xho*I, M1: *EcoR*I and *Xho*I (Additional file 2: Figure S2b, d). The nucleotide sequences of the CsTP 22.3 (accession number: EF077216.1) and M1 genes (accession number: FJ966085) were found to be identical to the previously published sequences by DNA sequencing.

### Production and characterization of VLPs

**Fig. 1:**
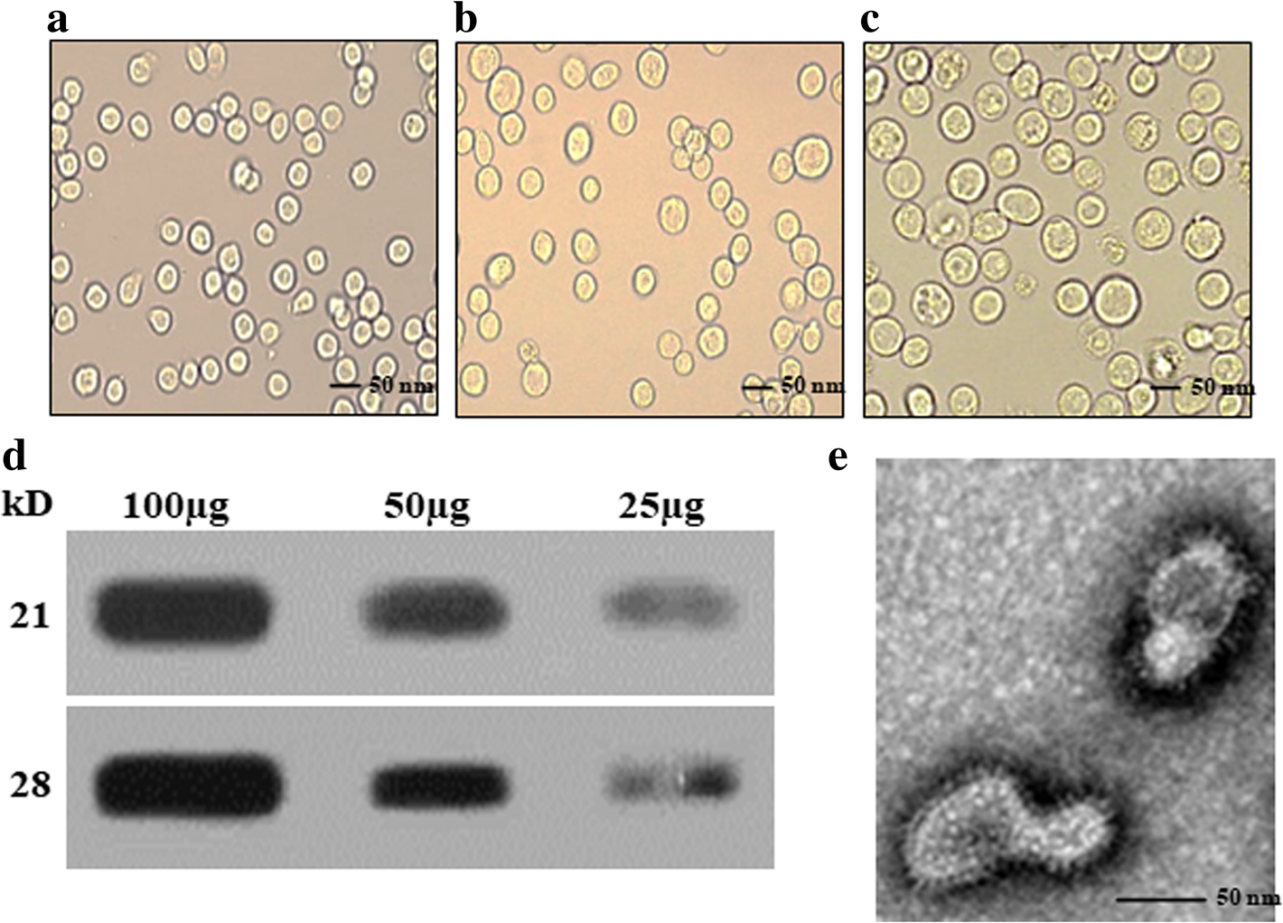
Recombinant baculovirus and virus-like particles. Baculoviruses expressing CsTP 22.3 or M1 were created (**a-c**), and virus-like particles (VLPs, **d**, **e**) expressing CsTP 22.3 together with influenza M1 were produced. **a** Naïve SF9 cells. **b** SF9 cells infected with baculovirus expressing influenza M1. **c** SF9 cells infected with baculovirus expressing CsTP 22.3. VLPs were characterized by western blots (**d**) and electron microscopy (**e**). The spikes representing CsTP 22.3 were observed on the surface of VLPs

VLPs were produced by co-infected CsTP 22.3 with influenza M1 recombinant baculoviruses (Fig. 1a-c). The components of generated VLPs were confirmed by western blot (Fig. 1d). VLPs were reacted with rat sera that were infected with *C. sinensis*. Influenza M1, used as VLPs core protein, was detected by monoclonal mouse anti-M1 antibody. VLPs exhibited spherical shapes under microscopy and spikes were observed on the surface of the VLPs (Fig. 1e).

### VLPs immunization induced humoral immunity

**Fig. 2:**
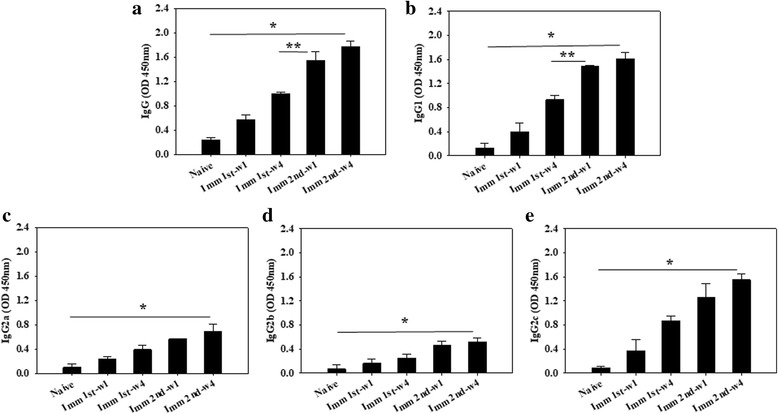
*Clonorchis sinensis*-specific IgG, IgG1, Ig2a, IgG2b and IgG2c antibody responses in rats immunized with VLPs. *Clonorchis sinensis*-specific IgG (**a**), IgG1 (**b**), IgG2a (**c**), IgG2b (**d**) and IgG2c (**e**) antibody responses were determined in the sera at weeks 1 and 4 from primary (Imm 1st-w1) and secondary immunization (Imm 2nd-w4). Significantly higher levels of IgG, IgG1, IgG2a and IgG2c antibody responses were observed after boost compared to those after prime (*P < 0.05, **P < 0.01)

**Fig. 3:**
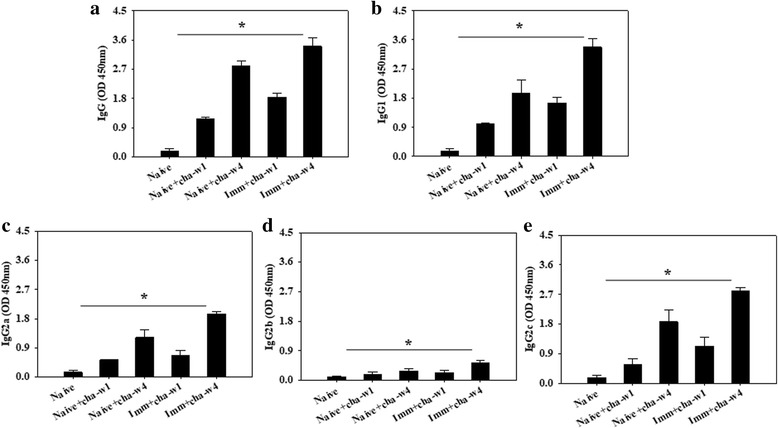
*Clonorchis sinensis*-specific IgG, IgG1, IgG2a, IgG2b and IgG2c antibody responses in VLP-immunized rats upon challenge infection. *Clonorchis sinensis*-specific IgG (**a**), IgG1 (**b**), IgG2a (**c**), IgG2b (**d**) and IgG2c (**e**) antibody responses were determined in the sera at weeks 1 and 4 from primary (Immu1st-w1) and secondary immunization (Immu2nd-w4). Significantly higher levels of IgG, IgG1, IgG2a and IgG2c antibody responses were observed at weeks 1 and 4 in immunized rats after challenge infection (*P < 0.05)

To evaluate *C. sinensis*-specific serum antibody responses induced by VLPs immunization, the *C. sinensis*-specific total IgG, IgG1, IgG2a, IgG2b and IgG2c antibody levels were determined at week 1 and 4 after prime and boost (Fig. 2; significant differences: IgG: *χ* ^2^ = 7.2, *df* = 2, *P* = 0.0273 and *χ* ^2^ = 9.8462, *df* = 2, *P* = 0.0073; IgG1: *χ* ^2^ = 6.4889, *df* = 2, *P* = 0.039 and *χ* ^2^ = 9.7124, *df* = 2, *P* = 0.0087; IgG2a: *χ* ^2^ = 6.4, *df* = 2, *P* = 0.041; IgG2b: *χ* ^2^ = 6.377, *df* = 2, *P* = 0.044; IgG2c: *χ* ^2^ = 7.2574, *df* = 2, *P* = 0.0298) and after challenge infections (Fig. 3; significant differences: IgG: *χ* ^2^ = 7.2, *df* = 2, *P* = 0.0273; IgG1; *χ* ^2^ = 6.477, *df* = 2, *P* = 0.038; IgG2a: *χ* ^2^ = 6.377, *df* = 2, *P* = 0.044; IgG2b: *χ* ^2^ = 6.4, *df* = 2, *P* = 0.041; IgG2c: *χ* ^2^ = 7.2, *df* = 2, *P* = 0.0273). IgG and IgG isotype responses were shown higher upon challenge infection compared to those before challenge infection (Figs. 2, 3). Significantly higher levels of *C. sinensis*-specific IgG, IgG1, IgG2a, IgG2b and IgG2c responses from rats immunized with VLPs were observed after boost, compared to those after prime (Fig. 2a-e). Significantly higher levels of *C. sinensis*-specific IgG, IgG1, IgG2a and IgG2c isotype responses were found at week 4 compared to those at week 1 upon prime (Fig. 2a-c, e). Importantly, *C. sinensis*-specific IgG1 and IgG2c antibody isotypes increased significantly, compared to IgG2a and IgG2b antibody isotypes (Fig. 2b-e). Antibody responses in the sera were also observed in immunized rats upon challenge infection. As shown in Fig. 3, significantly higher levels of *C. sinensis*-specific IgG, IgG1, IgG2a and IgG2c antibodies were detected at weeks 1 and 4 in immunized rats, compared to those unimmunized naïve rat controls (Fig. 3a-c, e). Higher levels of *C. sinensis*-specific IgG, IgG1, IgG2a and IgG2c antibody responses were found at week 4, compared to those at week 1 (Fig. 3a-c, e). Lower IgG2b antibody responses were determined compared to IgG1, IgG2a and IgG2c antibodies, in which IgG2b antibody response was higher at week 4, compared to that at week 1 (Fig 3d). These results indicate that VLPs induced strong humoral immune responses with induction of IgG1-, IgG2a- and IgG2c-dominant antibody responses, indicating VLP immunization induced Th1- and Th2-like responses. IgG, IgG1, IgG2 and IgG2c antibody responses were even higher after challenge infection, compared to those before challenge infection, which might contribute to the protection in VLPs-immunized rats.

### VLPs immunization induced significantly higher levels of mucosal IgG and IgA antibody responses

**Fig. 4:**
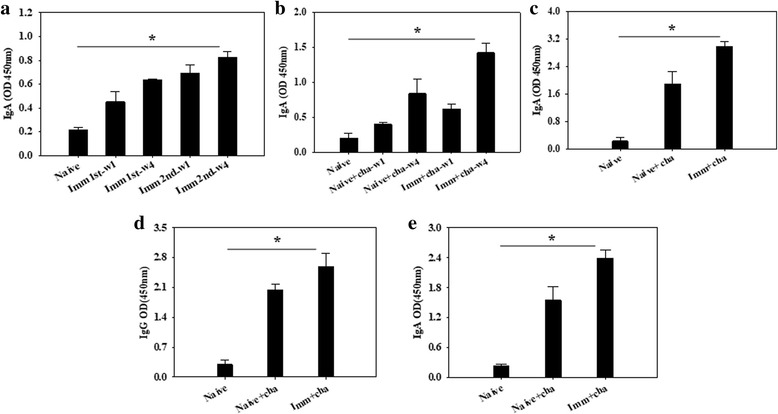
Mucosal IgA or IgG antibody responses in the feces, intestine and liver. Rat feces before and after challenge infections, and intestines and livers after challenge infection were collected to determine mucosal IgA or IgG antibody responses. Significantly higher levels of IgA antibody responses in the feces before (**a**) and after (**b**) challenge infection, in the intestine after challenge infection (**c**) and IgG and IgA antibodies in the liver after challenge infection (**d**, **e**) were observed at weeks 1 and 4 in immunized rats after challenge infection (*P < 0.05)

Rats were intranasally immunized with VLPs and challenge infected with *C. sinensis* metacercariae. To determine whether mucosal immunity can be induced, rat feces and intestines were collected before and after challenge infections. As shown in Fig. 4a, b, significantly higher levels of IgA antibodies were observed in feces, in which IgA antibody responses were higher after challenge infection (Fig. 4b, *χ* ^2^ = 8.0563, *df* = 2, *P* = 0.0178), compared to that before challenge infection (Fig. 4a; significant differences: *χ* ^2^ = 8.1563, *df* = 2, *P* = 0.012,). Intestine IgA antibody responses also were much higher in immunized rats compared to unimmunized control after challenge infection (Fig. 4c; significant differences: *χ* ^2^ = 7.2, *df* = 2, *P* = 0.0273). Liver IgG and IgA antibody responses were much higher, compared to those before challenge infections (Fig. 4d, e; significant differences: *χ* ^2^ = 6.1434, *df* = 2, *P* = 0.032; *χ* ^2^ = 6.5, *df* = 2, *P* = 0.04). These results indicate that intranasal immunization with VLPs in rats induced significantly higher levels of mucosal IgG and IgA antibody responses, which might also contribute to the protection in immunized rats.

### VLPs immunization induced antibody secreting cell response

**Fig. 5:**
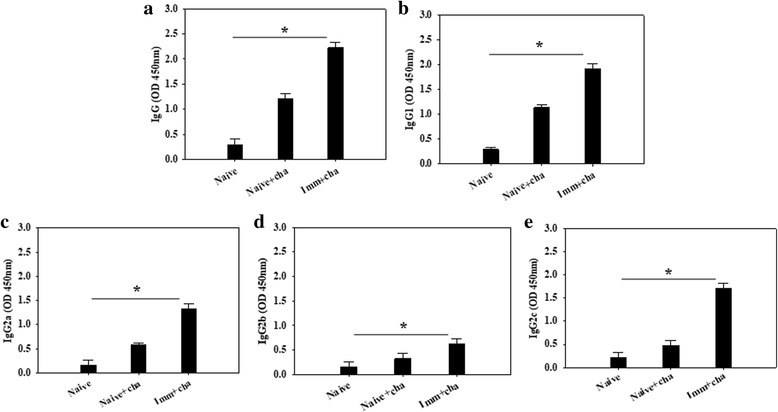
Antibody secreting cell responses in the spleen. Immunized rats were challenge infected with *C. sinensis* metacercariae at week 4 after boost and antibody-secreting cells (ASC) in the spleen were determined at week 4 after challenge infections. IgG (**a**), IgG1 (**b**), IgG2a (**c**), IgG2b (**d**) and Ig2C (**e**) antibody-secreting cells were seen as indicated (*P < 0.05)

To determine the antibody-secreting cell responses after challenge infection with *C. sinensis*, spleen cells were collected from rats and subjected to in vitro culture. Significantly higher levels of IgG, IgG1, IgG2a and IgG2c antibodies specific to *C. sinensis* ES Ag were secreted into culture supernatants by spleen cells of immunized rats than from cells derived from unimmunized rats (Fig. 5; significant differences: IgG: *χ* ^2^ = 8.5376, *df* = 2, *P* = 0.0135; IgG1: *χ* ^2^ = 8.5376, *df* = 2, *P* = 0.0135; IgG2a: *χ* ^2^ = 7.1997, *df* = 2, *P* = 0.027; IgG2b: *χ* ^2^ = 6.4889, *df* = 2, *P* = 0.039; IgG2c: *χ* ^2^ = 7.2, *df* = 2, *P* = 0.0237). Taken together, these results indicate that generated memory B cells from VLP immunized rats have the capacity to rapidly differentiate into antibody-secreting cells upon infection with *C. sinensis*.

### VLPs immunization induced CD4+ and CD8+ T cell response

**Fig. 6:**
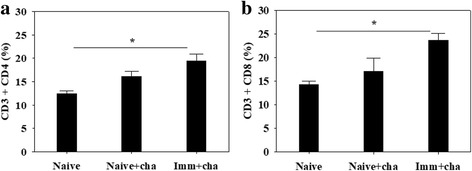
T cell responses. Immunized rats were challenge infected and at week 4 after challenge infections rats were sacrificed. CD4+ T cell (**a**) and CD8+ T cell (**b**) populations in the spleen were determined (*P < 0.05)

To determine CD4+ and CD8+ T cells in the spleen, cells were stained with CD4+ and CD8+ markers. As shown in Fig. 6a, b, both CD4+ T cells and CD8+ T cells were raised from immunized rats upon challenge infections, compared to non-immunized control rats (Fig. 6; significant differences: CD4+: *χ* ^2^ = 8.1723, *df* = 2, *P* = 0.0163; CD8+: *χ* ^2^ = 6.4733, *df* = 2, *P* = 0.0319).

### VLPs immunization significantly reduced inflammation responses in the liver

**Fig. 7:**
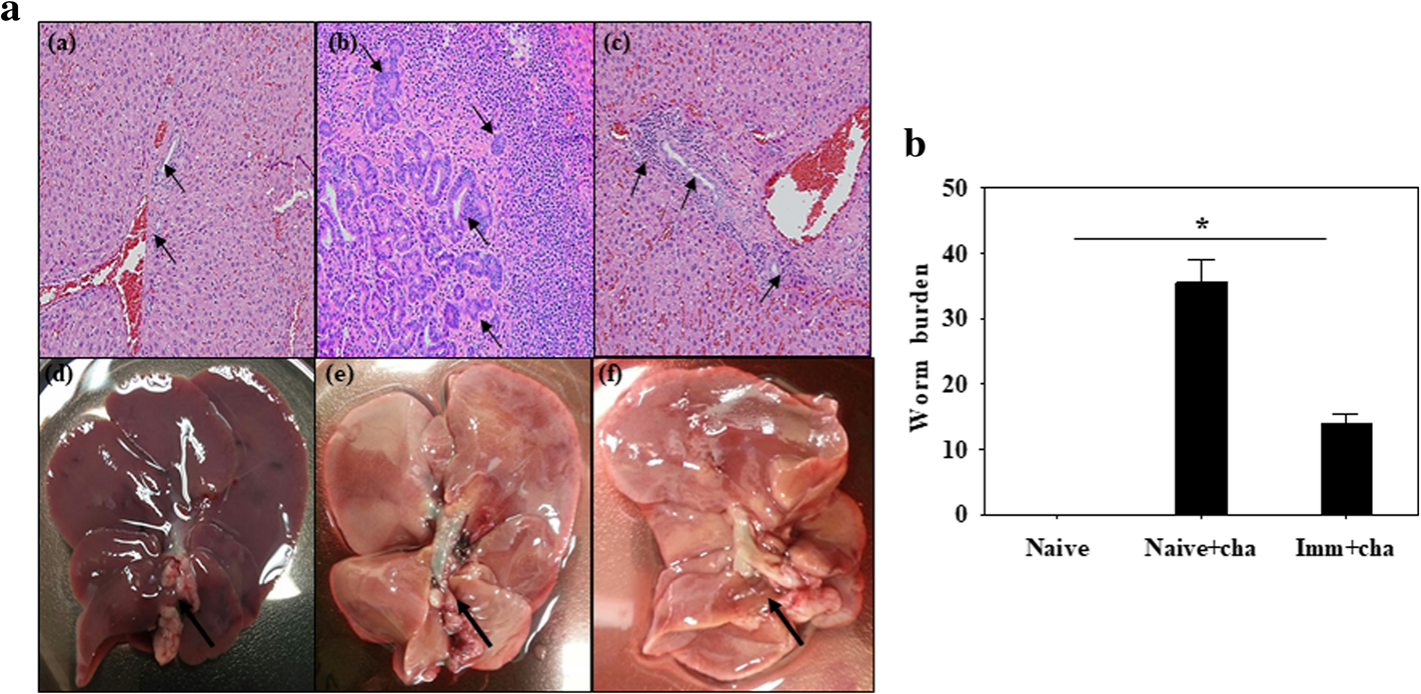
Inflammatory responses and worm burden in the liver. Liver tissues were collected from individual rats at week 4 after challenge, and tissue sections were stained with hematoxylin and eosin to assess liver inflammatory responses (200× magnification). Heavier periductal infiltration of inflammatory cells, such as plasma cells, lymphocytes and mononuclear cells was found in unimmunized rats upon challenge infection (**b**) compared to naïve rats (**a**) (**a** *vs* **b**). VLPs immunized rats showed less infiltration of inflammatory cells (**c**) compared to unimmunized rats upon challenge infection (**b**) (**c** *vs* **b**). Unimmunized (**e**), naïve (**d**) and immunized rats (**f**) showed different observed by naked eye. *Clonorchis sinensis* worm burden were determined from rats immunized with VLPs and controls (**b**). Liver tissues were collected from individual rats at week 4 after challenge. Rats immunized with VLPs showed significantly lower levels of worm burden compared to naïve control upon challenge infection (**b**, *P < 0.05)

Histopathological change upon *C. sinensis* infection was assessed to evaluate inflammation status in the liver. As seen in Fig. 7a, heavier periductal infiltration of inflammatory cells, such as plasma cells, lymphocytes and mononuclear cells, was found in unimmunized rats upon challenge infection [Fig. 7a(a) compared to naïve rats (Fig. 7a(b)]. Notably, VLPs immunized rats [Fig. 7a(c)] showed less infiltration of inflammatory cells compared to unimmunized naïve rats upon challenge infection. Unimmunized naïve rats showed significantly enlarged liver duct upon challenge [Fig. 7a(d) compared to naïve (Fig. 7a(e)) and immunized (Fig. 7a(f))]. These results indicate that VLPs immunization significantly reduced liver inflammation following *C. sinensis* metacercariae challenge infection.

### VLPs immunization significantly reduced liver *C. sinensis* worm loads following challenge infection

Liver adult worm loads following infection are the most prominent indicator to assess vaccine protective efficacy. Immunized and naïve rats were infected with *C. sinensis* metacercariae (50/rat) at 4 weeks after boost, and liver worm burden was determined at week 4 post-infection. As shown in Fig. 7b (significant differences: *t*_(44)_ = 4.46, *P* = 0.021), significantly decreased worm loads were detected in VLPs immunized rats compared to unimmunized rat control (70.2%). The result indicates that VLPs immunization effectively reduced worm burden in the liver.

## Discussion

*Clonorchis sinensis* epidemics are one of the most common zoonoses and remain a major health concern as *C. sinensis* infection is closely related to cholangiocarcinoma (CCA), fibrosis and other human hepatobiliary diseases [6]. However, protective immunity against *C. sinensis* infection remains unknown. Study of vaccines against *C. sinensis* infection would have a significant impact. Virus-like particles vaccines have been successful in their safety and efficacy in virus fields in preclinical and clinical studies [16]. Commercial VLPs vaccines against hepatitis B virus (HBV) and human papilloma virus (HPV) have been escalated the interests in developing new VLP vaccines against respiratory viruses. Recently, the VLPs against parasite infections of *Trichinella spiralis* and *Toxoplasma gondii* showed encouraging results [26, 27]. The current study investigated for the first time the protective efficacy of VLPs containing the *C. sinensis* tegumental protein 22.3 kDa (CsTP 22.3) and the influenza matrix protein (M1) as a core protein. We found that intranasal immunization of VLPs vaccine induced *C. sinensis*-specific IgG, IgG2a and IgG2c in the sera and IgA responses in feces and intestines. Vaccinated rats showed less inflammatory responses in the liver compared to unvaccinated controls. Notably, significantly lower adult worm loads (70.2%) were measured in the liver of rats immunized with VLPs, compared to those of naïve rats, providing partial protection against *C. sinensis* infection.

The tegument of *C. sinensis* is critically important in identifying potential antigens for diagnosis and vaccine candidates [14]. Recombinant *C. sinensis* tegumental protein 22.3 kDa has been shown to confer protection against *C. sinensis*, reducing worm burden by 44.7% upon challenge infection with *C. sinensis* metacercariae [14]. In the current study, we developed baculovirus expressing CsTP 22.3, and generated virus-like particles containing CsTP22.3. The VLPs we generated exhibited spherical shapes, and CsTP 22.3 protein exhibited as spikes on VLP surface under electron microscopy (Fig. 1e). Influenza M1 as a core protein in VLPs resembles virions in morphology and size. VLPs contain repetitive, high density displays of *C. sinensis* surface protein CsTP22.3 that seem to elicit strong T and B cell immune responses [26]. *Bacillus subtilis* spores displaying TP22.3 elicited TP22.3-specific IgA mucosal immunity, showing 44.7% of protection against *Clonorchis sinensis* [14]. However, in our current study, VLPs expressing CSTP22.3 protein provided systemic IgG and mucosal IgA antibodies, as well as T cell immune responses, resulting in higher protection. Thus, VLPs vaccination provided higher vaccine efficacy (70.2%) than the previous report.

Induction of mucosal immunity is critical in inducing protection against pathogens in the mucosal organ. Intranasal immunization with inactivated influenza virus (PR8) elicited mucosal IgA antibodies in mucosal organs. Significantly higher levels of virus-specific IgA antibodies were detected in the lung, vagina and intestine (feces), compared to those before immunization [28]. The results indicate that intranasal immunization induced virus-specific IgA antibodies in respiratory tract at the site of vaccination route, as well as the reproductive organs and gastrointestinal tract. Since *C. sinensis* infection occurs through the mucosa of the gastrointestinal tract, we hypothesize that intranasal immunization route applied in current study will induce mucosal antibody response. As indicated in current study, VLPs vaccinated rats showed significantly higher levels of *C. sinensis*-specific IgA antibodies in feces and intestine, compared to unimmunized control rats (Fig. 4). Interestingly, after challenge infection, significantly higher levels of *C. sinensis*-specific IgG or/and IgA antibodies in feces and livers were rapidly expanded compared to those before challenge infection (Fig. 4a-e). Thus, enhanced mucosal IgG and IgA antibodies might be involved in the protection induced by VLP vaccination. Mucosal IgG and IgA antibodies in the bile ducts of liver are known to be associated with the resistance against *C. sinensis* in rats [20, 29].

Rats have four subclasses known as IgG1, IgG2a, IgG2b and IgG2c; IgG1 and IgG2a are Th2-related, and IgG2b and IgG2c are Th1-related [30]. Our analysis of IgG isotypes IgG1, IgG2a, IgG2b and IgG2c antibodies in the sera and isotype antibody-secreting plasma cells in the spleen in VLPs vaccinated rats showed that significantly higher levels of IgG1 and IgG2c antibodies were observed, compared to IgG2a and IgG2b (Figs. 2, 3, 5) in the sera as well as in the spleen, indicating that VLPs vaccination induced Th1- and Th2-related isotypes.

To investigate other potential components contributing to protection, we determined CD4+ and CD8+ T cells in the spleen. We found that VLP vaccination induced higher levels of CD4+ and CD8+ T cell responses, which might also contribute to the protection against *C. sinensis* infection. These results are consistent with previous studies showing that significantly higher levels of CD4+ and CD8+ T cell responses induced by vaccination with *T. gondii* IMC VLPs may be involved in the protection against *T. gondii* infection [27].

In summary, our results demonstrate that *C. sinensis* VLPs containing CSTP 22.3 can induce systemic and mucosal IgG, IgA antibodies and CD4+ and CD8+ T cell responses, which can reduce worm burden in the bile duct. These results provide evidence that the VLP format is highly immunogenic and is a promising approach for developing effective prophylactic vaccines.

## Conclusions

Our results demonstrate that VLP vaccine containing *C. sinensis* CsTP 22.3 tegumental can induce humoral and cellular immune responses, resulting in partial protection against *C. sinensis* infection. Interestingly, mucosal IgA antibody and IgG, IgG1, IgG2a, IgG2c antibody responses induced by immunization were increased upon challenge infection. These results provide an effective approach for developing vaccines based on VLPs for protection against the *C. sinensis*.

## Additional files

Additional file 1: Figure S1. *Clonorchis sinensis* ES product was separated by sodium dodecyl sulphate-polyacrylamide gel electrophoresis (SDS-PAGE) in 12% polyacrylamide gels using a Mini-PROTEAN Tetra Cell electrophoresis unit (Bio-Rad, Herculus, USA). *Clonorchis sinensis* ES product (40, 20, 10, 5 μg) was loaded and incubated at 150 V for 1 h. To determine the proteins in *C. sisnensis* ES product, the gel was stained with Coomassie blue. *Clonorchis sienensis* CsTP22.3 protein was detected in *C. sinensis* ES product. (TIFF 119 kb)

Additional file 2: Figure S2. *Clonorchis sinensis* CsTP 22.3 and influenza M1 genes were PCR amplified (**a**, **c**) and cloned into PFastBac vector (**b**, **d**). *Abbreviations*: M, marker; CsTP, *C. sinensis* CsTP 22.3 gene; M1, influenza M1. (TIFF 123 kb)

## Abbreviations

CsTP 22.3: Tegumental protein 22.3 kDa
ELISA: Enzyme-linked immunosorbent assay
ES Ag: Excretory-secretory antigen
HRP: Horseradish peroxidase
PBS: Phosphate buffered saline
SDS-PAGE: Sodium dodecyl sulphate-polyacrylamide gel electrophoresis
VLP: Virus-like particle
