# Supplementary figures and images for: Virus-like particles vaccine containing Clonorchis sinensis tegumental protein induces partial protection against Clonorchis sinensis infection
Source: Parasit Vectors. 2017 Dec 29;10:626. doi: 10.1186/s13071-017-2526-5 (PMC5747077; doi:10.1186/s13071-017-2526-5)

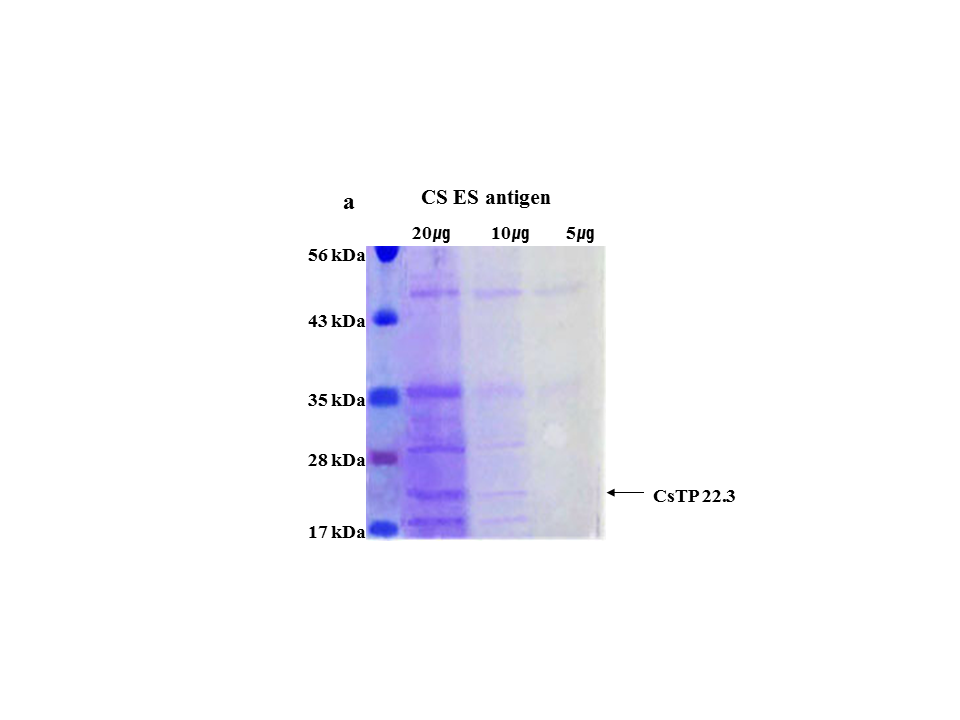

Supplement: Supplementary file 1 — Clonorchis sinensis ES product was separated by sodium dodecyl sulphate-polyacrylamide gel electrophoresis (SDS-PAGE) in 12% polyacrylamide gels using a Mini-PROTEAN Tetra Cell electrophoresis unit (Bio-Rad, Herculus, USA). Clonorchis sinensis ES product (40, 20, 10, 5 μg) was loaded and incubated at 150 V for 1 h. To determine the proteins in C. sisnensis ES product, the gel was stained with Coomassie blue. Clonorchis sienensis CsTP22.3 protein was detected in C. sinensis ES product. (TIFF 119 kb) [file 13071_2017_2526_MOESM1_ESM.tif]

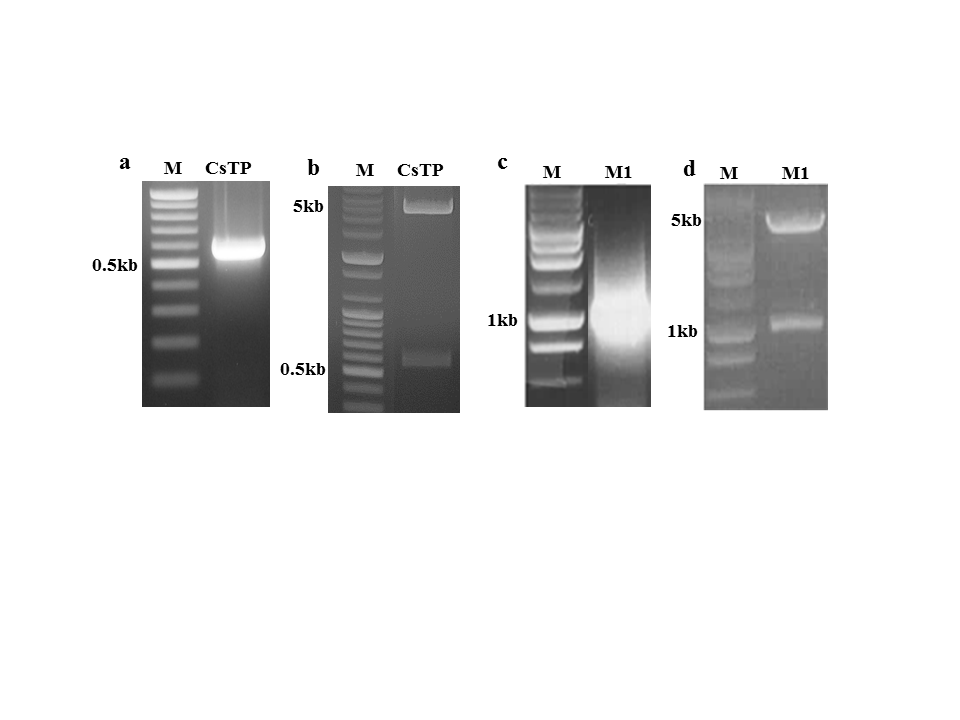

Supplement: Supplementary file 2 — Clonorchis sinensis CsTP 22.3 and influenza M1 genes were PCR amplified (a, c) and cloned into PFastBac vector (b, d). Abbreviations: M, marker; CsTP, C. sinensis CsTP 22.3 gene; M1, influenza M1. (TIFF 123 kb) [file 13071_2017_2526_MOESM2_ESM.tif]
